# Impact of commonly prescribed exercise interventions on platelet activation in physically inactive and overweight men

**DOI:** 10.14814/phy2.12951

**Published:** 2016-10-17

**Authors:** Andrew Haynes, Matthew D. Linden, Elisa Robey, Gerald F. Watts, Hugh Barrett, Louise H. Naylor, Daniel J. Green

**Affiliations:** ^1^ School of Sport Science, Exercise and HealthUniversity of Western AustraliaCrawleyWestern AustraliaAustralia; ^2^ School of Pathology and Laboratory MedicineUniversity of Western AustraliaCrawleyWestern AustraliaAustralia; ^3^ Cardiometabolic ServicesDepartment of CardiologyRoyal Perth HospitalWestern AustraliaAustralia; ^4^ School of Medicine and Pharmacology Royal Perth Hospital UnitUniversity of Western AustraliaCrawleyWestern AustraliaAustralia; ^5^ Research Institute for Sport and Exercise ScienceLiverpool John Moores UniversityLiverpoolUnited Kingdom; ^6^ Principal Research FellowNational Health and Medical Research CouncilCanberraACTAustralia

**Keywords:** Acute coronary syndromes, physical activity, thrombosis

## Abstract

The exercise paradox infers that, despite the well‐established cardioprotective effects of repeated episodic exercise (training), the risk of acute atherothrombotic events may be transiently increased during and soon after an exercise bout. However, the acute impact of different exercise modalities on platelet function has not previously been addressed. We hypothesized that distinct modalities of exercise would have differing effects on *in vivo* platelet activation and reactivity to agonists which induce monocyte‐platelet aggregate (MPA) formation. Eight middle‐aged (53.5 ± 1.6 years) male participants took part in four 30 min experimental interventions (aerobic AE, resistance RE, combined aerobic/resistance exercise CARE, or no‐exercise NE), in random order. Blood samples were collected before, immediately after, and 1 h after each intervention, and incubated with one of three agonists of physiologically/clinically relevant pathways of platelet activation (thrombin receptor activating peptide‐6 TRAP, arachidonic acid AA, and cross‐linked collagen‐related peptide xCRP). In the presence of AA, TRAP, and xCRP, both RE and CARE evoked increases in MPAs immediately post‐exercise (*P *<* *0.01), whereas only AA significantly increased MPAs immediately after AE (*P *<* *0.01). These increases in platelet activation post‐exercise were transient, as responses approached pre‐exercise levels by 1 h. These are the first data to suggest that exercise involving a resistance component in humans may transiently increase platelet‐mediated thrombotic risk more than aerobic modalities.

## Introduction

Monocyte‐platelet aggregates (MPAs) result from the interaction between activated platelets and monocytes. MPAs are considered a more sensitive marker of platelet activation in humans than platelet surface CD62P expression, which is rapidly cleaved from the platelet surface and has a lower detection sensitivity by flow cytometry (Michelson et al. 2001). Platelets become activated as a consequence of exposure to various in vivo agonists including thrombin, collagen, and the arachidonic acid (AA) by‐product thromboxane A2. Each of these physiological agonists targets a different platelet surface receptor and therefore activates platelets via independent pathways (Clemetson and Clemetson 2013). Atherothrombosis is largely a platelet‐mediated event (Davi and Patrono 2007), and MPAs are elevated in patients with stable coronary artery disease (Furman et al. 1998) and acute myocardial infarction (Furman et al. 2001).

Despite the well‐established health benefits of long‐term exercise (Blair and Morris 2009), it is acknowledged that the risk of an atherothrombotic event is transiently increased in the period during and immediately following an acute exercise bout (Thompson et al. 2007), a phenomenon referred to as the “exercise paradox” (Maron 2000). While some evidence suggests there is a positive association between the risk of acute coronary syndromes (ACS) and exercise intensity (Mittleman et al. 1993), little is known regarding the implications of different modalities of exercise on transient atherothrombotic risk and MPA formation. Three modes of exercise that are commonly utilized in humans are steady‐state aerobic exercise (AE; walking, running, cycling), resistance exercise (RE; weight lifting) and a combination of aerobic and resistance exercise (CARE; circuit training). These modalities are prescribed to achieve functional improvements in cardiorespiratory fitness and/or skeletal muscle strength and endurance in both primary and secondary prevention settings (Bjarnason‐Wehrens et al. 2004; Garber et al. 2011). They are also widely utilized in the general community in the context of personal health and fitness training.

While the acute and chronic effects of AE, RE and CARE on skeletal muscle and the cardiovascular system have previously been documented (George et al. 2011; Kraemer et al. 1995; Spence et al. 2013, 2011), no previous study has directly compared the impact of these distinct modes of exercise on MPAs. Since AE and RE have distinct impacts on the cardiovascular system and skeletal muscle (Bacchi et al. 2012; Bloomer et al. 2005; Drummond et al. 2005; Fowler et al. 2013; Heffernan et al. 2007), we hypothesized that they may confer divergent impacts on platelets. The aim of this study was to investigate the acute impact of different exercise modalities on MPAs in humans, including reactivity of platelets to a variety of relevant physiological platelet agonists. We hypothesized that each modality of exercise would have different impacts on MPA formation.

## Materials and Methods

A sample size calculation was conducted for paired samples indicating that as few as three participants would be sufficient to detect a 6% difference in MPAs with an *α* of 0.05 (Rosner 1995).

### Ethical approval

All procedures adhered to the Declaration of Helsinki and were approved by the Human Research Ethics Committee of The University of Western Australia. All participants provided written, informed consent prior to any procedures being undertaken.

### Participants

Eight apparently healthy male participants aged 40–65 years with no previous evidence of cardiovascular disease were recruited from the local community in Perth, Australia by poster advertisements placed in public places. Individuals were defined as physically inactive following completion of the International Physical Activity Questionnaire, taking part in <1  h of structured physical activity a week, and exercise naïve in that they do not take part in any form of regular exercise training. Those taking prescription medications, or with musculoskeletal injuries that would hinder their ability to exercise were excluded. As part of the screening process, an exercise stress test was performed in the presence of a physician following standard guidelines (Pescatello 2014) and individuals with any evidence of exercise‐induced ischemia or arrhythmia were excluded from further participation. Although apparently healthy, participants exhibited the following risk factors associated with cardiometabolic disease: advancing age, physical inactivity, abdominal obesity, and raised blood lipid levels. Fasting glucose and full lipid profile blood tests were conducted, as was a dual‐energy X‐ray absorptiometry (DEXA) scan to illustrate the general characteristics of the participants included in the study.

### Preliminary sessions

Participants were familiarized with equipment, exercises, and procedures to be included in subsequent sessions. In two separate visits, participants completed a maximal exercise test on a cycle ergometer, and repetition maximum (RM) strength tests for the six resistance exercises listed below. Percentages of maximum were used to regulate and standardize the intensity of exercise used in subsequent sessions, according to well‐established principles of exercise physiology (Garber et al. 2011).

### Experimental sessions

All participants completed four experimental sessions, in random order, with each separated by at least 7 days, using a repeated‐measures crossover design. These included 30 min of: AE, RE, CARE, or no‐exercise (NE). A standardized stretching routine was included in the warm‐up of all exercise sessions composed of a combination of static and dynamic stretches targeting all major muscle groups.

Participants arrived at the laboratory in the morning around 8 am, having being asked to abstain from caffeine consumption for 12 h and alcohol for 24 h. All consumed the same carbohydrate‐based breakfast including toast or cereal, avoiding fruit, vegetables, and meat products. Participants were specifically asked about drug use and none used anti‐inflammatory, aspirin‐containing or other medications that affect platelet or leukocyte function. Adherence to the protocol was confirmed by questionnaire on arrival to each session.

Participants then began a 20 min period of quiet rest in a semirecumbent position, prior to the first blood collection. This was followed by the (no) exercise activity that was to be undertaken on that day. Following each exercise session, participants resumed a semirecumbent position and remained there for 1 h. Heart rate was monitored during all exercise (Polar RS300X, Polar Electro Oy, Finland).

### No exercise protocol

Participants lay on a bed in a semirecumbent position for the duration of the no‐exercise session, which lasted approximately 2.5 h.

### Aerobic exercise protocol

Participants completed a 4 min warm‐up on a rowing ergometer achieving 40% heart rate reserve (HRR) by the end of the fourth minute. The main exercise component included 13 min on a cycle ergometer (Circle Fitness, P&F Brothers Ind., Corp. Taiwan) at 65% Watts(W)max, followed by 13 min on a Concept 2 PM3 rowing ergometer (Concept 2 Inc., Morrisville, VT). Heart rate monitoring ensured a steady‐state was achieved and intensity on the rowing ergometer was matched to the heart rate during cycling. Heart rate, ratings of perceived exertion (RPE) using Borg's 6–20 scale (Borg 1970) and absolute intensity were documented at minutes 5, 10, and 13 of bicycle and rowing ergometry. This protocol was designed to replicate a typical AE session used in a gymnasium setting (Garber et al. 2011).

### Resistance exercise protocol

Three sets of each resistance exercise were completed. A 120 sec time period was allocated for each set, made up of 40 sec working and 80 sec recovery. Firstly, participants completed one set of each exercise with a resistance of 40% 1 RM, followed by two consecutive sets of each exercise at 65% 1 RM. Exercise stations included (1) sitting cable chest press, (2) leg press, (3) lateral pull‐down, (4) cable shoulder press, (5) sitting machine hamstring flexion, and (6) cable bicep curl with a rope attachment. Repetitions were continued for the entire 40 sec working period or until muscular failure, whichever came first. Heart rate, RPE, and the number of repetitions completed were documented immediately following the last repetition of each set. As for the AE session described above, we adopted an ecologically valid approach to the design of this session, which closely resembles typically prescribed RE.

### Combined aerobic and resistance exercise protocol

The CARE training session included all the exercises that made up the AE and RE sessions, but with shorter recovery times between RE stations so that total exercise time was matched to the other two experimental conditions. Participants completed three 10 min circuits; the first circuit at an intensity of 40% maximum (i.e., Wmax for AE, 1 RM for RE), and the following two circuits at 55% maximum. Each exercise circuit consisted of six RE and two AE, performed in the order: three RE (sitting cable chest press, leg press, lateral pull‐down), one AE (bicycle ergometer), three RE (cable shoulder press, sitting machine hamstring flexion and cable bicep curl with a rope attachment), one AE (rowing), with 2 min recovery between each circuit. Resistance exercises were allocated 60 sec (40 sec working and 20 sec transition time) and aerobic exercises were allocated 120 sec (100 sec working and 20 sec transition time). Heart rate, RPE, and repetitions completed for resistance exercises were recorded at the final moments of exercise at each station.

### Blood collection

Venous blood was collected by separate venepunctures from the antecubital region at three time‐points during each session; (1) before exercise (2) after exercise, and (3) 1 h post‐exercise (identical time‐intervals during NE), using a 21G winged needle set (Greiner bio‐one) with minimal stasis (Harrison et al. 2011). The first 2 mL of blood was collected into a nonadditive discard tube followed by a 4 mL 3.2% sodium citrate tube (Vacuette by Greiner bio‐one).

### Monocyte‐platelet aggregates

Within 10 min of collection, blood was passed from the 3.2% sodium citrate tube and processed for the assessment monocyte‐platelet aggregation. Antibodies for CD14 (monocyte identifier) conjugated to the fluorophore Brilliant Violet (BV) 421 (Clone M5E2; BioLegend, San Diego, CA) and CD42b (platelet identifier) conjugated to Allophycocyanin (APC) (Clone HIP1; BioLegend) or IgG isotype control (BioLegend) were used. For each blood collection, there were nine MPA reaction tubes; isotype control, no agonist, positive control and two concentrations of the following three agonists; thrombin receptor activating peptide‐6 (TRAP‐6) (SFLLRN, Sigma‐Aldrich, MO), AA (Sodium arachidonate, Bio/Data Corp., PA) and cross‐linked collagen‐related peptide (xCRP) (Farndale Lab, University of Cambridge, UK). Each reaction tube contained a total liquid volume of 80 *μ*L (20 *μ*L of undiluted whole blood, 40 *μ*L of antibody cocktail made up of saturating concentrations of CD14 BV421 and CD42b APC in HEPES saline buffer, and 20 *μ*L of agonist or HEPES saline buffer in the absence of agonist). An isotype control was included to account for nonspecific binding and a final concentration of 250 *μ*M TRAP was used as a positive control. Absence of spectral overlap was confirmed by single‐color comp bead controls (BD Biosciences).

Blood was added to the following concentrations of agonists; 2 *μ*m and 5 *μ*mol/L TRAP‐6, 10 *μ*g/mL and 125 *μ*g/mL AA and 200 ng/mL and 1 *μ*g/mL cross‐linked collagen‐related peptide. Agonist concentrations were determined by dose–response curve to identify submaximal and threshold concentrations, in order to demonstrate either up‐ or down‐regulation of platelet response to each. Samples were incubated at room temperature with the exception of tubes containing AA and xCRP, which were incubated at 37°C using a dry block heater (Ratek DBH20D, Victoria, Australia). Following exactly 15 min incubation, all samples were fixed and red cells lysed with 800 *μ*L of BD FACSLyse solution (BD Biosciences, CA). Samples were then stored at 4°C in the dark and analyzed by flow cytometry (BD FACSCanto^TM^ II, BD Biosciences) within 24 h at a low flow rate for 10 min per tube, to avoid coincident events (Hui et al. 2015).

Data output from flow cytometry was analyzed using FlowJo v.X software (FlowJo LLC., San Carlos, CA). A gating strategy was devised to eliminate leukocyte‐doublet events (Majumder et al. 2012). Monocytes were then identified by their characteristic forward and side scatter properties and differential CD14 expression. Monocyte‐platelet aggregates were identified by CD42b expression above isotypic control, and confirmed by positive control. MPAs were expressed as the percentage of the monocyte population.

### Statistics

Two concentrations (threshold and submaximal) of each agonist were used to detect both increased and decreased sensitivity of platelets to agonist exposure, respectively. These data were used to calculate area under the concentration curve for each agonist, at each time‐point (pre, post, 1 h‐post). All of the agonist‐induced MPA data, statistics and results are presented as area under the concentration curve (AUC) in arbitrary units for each agonist minus the no agonist.

Statistical analyses were performed using SPSS 22 (IBM, Armonk, NY) software. For data meeting the assumptions of parametric statistical tests, two‐way repeated measures ANOVA's were conducted to test for differences between modalities, across time and interaction of modality × time effects. If significance was found, multiple one‐way repeated measures ANOVA tests were conducted to determine where differences occurred within‐modality over time and for corresponding time‐points between modalities, with post hoc Tukey's least significant difference test. For data failing the assumptions of parametric tests, multiple nonparametric Friedman tests were conducted for within‐modality differences over time and for corresponding time‐points between the four different modalities. Statistical significance was assumed at *P *<* *0.05.

## Results

Eight men (53.5 ± 1.6 years) completed the study. Baseline characteristics are presented in Table 1. Data gained from the maximal aerobic exercise test included: HRmax 176 ± 4 bpm, time to exhaustion 11.3 ± 0.5 min, peak power output 173 ± 8 watts and Vo_2_ peak 32.5 ± 1.3 mL** **kg^−1^ ****min^−1^. Physiological data recorded during each of the exercise bouts (AE vs. RE vs. CARE) were as follows: HR bpm (%HRmax) 141 ± 2 bpm (80%) versus 122 ± 2 bpm (69%) versus 134 ± 1 bpm (76%) and RPE 14 ± 0 versus 17 ± 0 versus 15 ± 0.

**Table 1:** Baseline characteristics, anthropometric, dual‐energy X‐ray absorptiometry (DEXA) and biochemistry variables

Anthropometric data	
Height (cm)	173 ± 1.9
Body mass (kg)	86.2 ± 4.1
Body mass index (kg/m^2^)	28.8 ± 1.4
Waist girth (cm)	99.3 ± 2.1
Hip girth (cm)	105.7 ± 2.4
Waist/Hip ratio	0.94 ± 0.01
Resting HR	bpm
Heart rate	62 ± 2
Resting blood pressure	mmHg
Systolic BP	128 ± 4
Diastolic BP	82 ± 4
Mean arterial pressure	100 ± 4
DEXA	%
Total body fat	32.5 ± 1.6
Body fat legs	28.4 ± 1.9
Body fat trunk	38.2 ± 1.7
Body fat android	43.6 ± 1.9
Body fat gynoid	34.4 ± 1.9
Fasting biochemistry	mmol/L
Cholesterol	6.0 ± 0.3
Triglyceride	1.8 ± 0.2
LDL‐C	4.1 ± 0.3
HDL‐C	1.1 ± 0.1
Cholesterol/HDL ratio	5.5 ± 0.4
Glucose	5.2 ± 0.1

Data is mean ± SEM, *n* = 8.

HR, heart rate; BP*,* blood pressure; LDL‐C*,* low‐density lipoprotein cholesterol; HDL‐C high‐density lipoprotein cholesterol.

### Monocyte‐platelet aggregation with acute exercise

#### MPAs no agonist

In the absence of agonist stimulation, no significant difference was observed in the percentage of MPAs between the three time‐points within any experimental treatment (mean ± SEM, NE: pre 4.0 ± 0.7%, post 3.9 ± 0.6%, 1 h 4.4 ± 0.7%, *P *=* *0.197; AE: pre 3.8 ± 0.7%, post 3.7 ± 0.7%, 1 h 4.7 ± 1.3%, *P *=* *0.417; RE: pre 4.2 ± 0.7%, post 6.5 ± 2.0%, 1 h 4.2 ± 0.9%, *P *=* *0.093 and CARE: pre 4.1 ± 0.7%, post 5.0 ± 1.4%, 1 h 4.1 ± 0.9%, *P *=* *0.417). No significant difference was found between the four modalities at pre (*P *=* *0.615), post (*P *=* *0.740), and 1 h‐post (*P *=* *0.717) time‐points.

### Arachidonic acid

**Figure 1:**
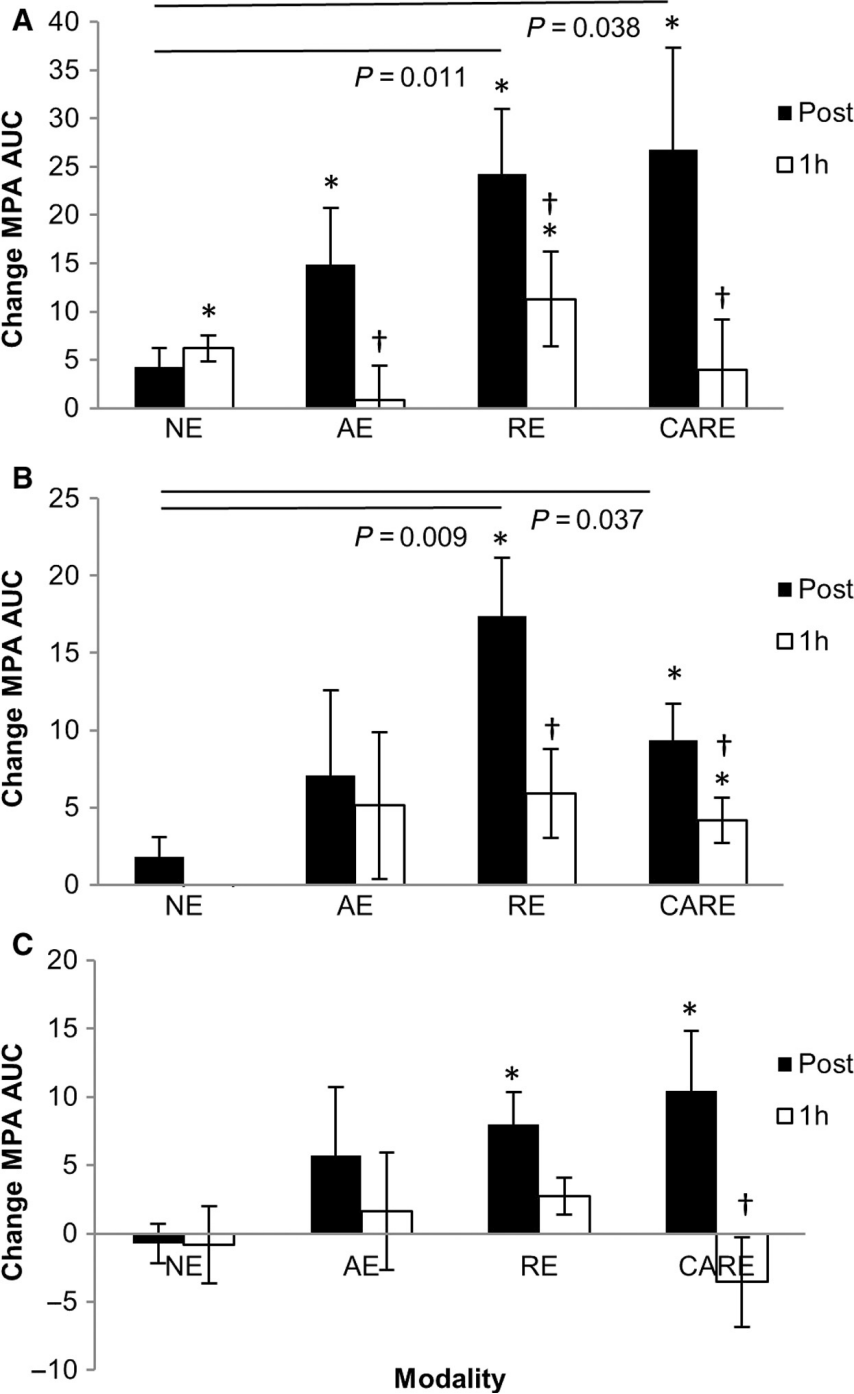
Change (mean ± SE) from the ‘pre’ time‐point (area under the curve) for agonist‐induced monocyte‐platelet aggregate formation. Blood collected pre, post, and 1 h post; no‐exercise (NE), aerobic exercise (AE), resistance exercise (RE) and combined aerobic and resistance exercise (CARE), and incubated with arachidonic acid (A), thrombin receptor activating peptide (B) and collagen‐related peptide (C). *signifies significant difference from pre, †signifies significant difference from post (*P *<* *0.05).

When blood samples were incubated with arachidonic acid, significant time (*P *=* *0.006) and time × modality interaction (*P *=* *0.003) effects were evident. As shown in the Figure 1 (panel A), AA stimulation significantly increased MPA area under the curve above pre‐exercise levels immediately post‐exercise under all three modalities (delta change ± SEM, AE: 14.9 ± 5.8 AUC, *P *=* *0.038; RE: 24.3 ± 6.7 AUC, *P *=* *0.009; CARE 26.8 ± 10.5 AUC, *P *=* *0.039). A duration of 1 h after cessation of exercise, MPAs significantly decreased from immediate post‐exercise levels for all modalities (delta decrease, AE; −14.0 AUC, *P *=* *0.006, RE; −13.0 AUC, *P *=* *0.018 & CARE; −22.8 AUC, *P *=* *0.009). However, MPAs were still elevated above pre‐exercise levels 1 h after RE (11.3 ± 4.9 AUC, *P *=* *0.05).

Comparison between modalities at corresponding time‐points indicated that no differences were found pre‐exercise (*P *=* *0.580) or 1 h post (*P *=* *0.468), but significant differences were present at the immediately post‐exercise time‐point (*P *=* *0.05). Tukey's post hoc test indicated that compared to no‐exercise (NE; 41.8 ± 8.7 AUC), MPAs were greater following RE (56.2 ± 10.0 AUC, *P *=* *0.011 vs. NE) and CARE (62.0 ± 11.0 AUC, *P *=* *0.038 vs. NE), but there was no difference between NE and aerobic (AE 49.8 ± 5.4 AUC, *P *=* *0.491 vs. NE). No significant differences were observed between the three exercise protocols.

### Thrombin receptor activating peptide‐6

In samples incubated with TRAP, there were significant main effects for time (*P *=* *0.005) and interaction between time and modality (*P *=* *0.049). As shown in the Figure 1 (panel B), both RE and CARE resulted in significant changes in TRAP‐induced MPAs over time (*P *=* *0.001 for each), with MPA AUC elevated above pre‐exercise levels immediately post‐exercise for RE and CARE (17.4 ± 3.7 AUC, *P *=* *0.002 and 9.4 ± 2.4 AUC, *P *=* *0.005), respectively. MPAs decreased from immediate post‐exercise levels following 1 hr recovery for both RE (−9.3 AUC, *P *=* *0.002) and CARE (−4.6 AUC, *P *=* *0.024). Following RE, MPAs at 1 h post‐exercise were not different to pre‐exercise levels (*P *=* *0.078), but were still elevated above pre‐exercise levels 1 h post‐CARE (4.2 ± 1.5 AUC, *P *=* *0.024). No effect was seen with NE or AE over time (*P *=* *0.440 and *P *=* *0.482, respectively).

A significant difference was found between modalities immediately post‐exercise (*P *=* *0.035) and was due to elevated MPAs following RE and CARE compared to NE (RE vs. NE, *P *=* *0.009) (CARE vs. NE, *P *=* *0.037). AE was nonsignificantly elevated at this time‐point (AE 7.1 ± 5.5 AUC vs. NE 1.8 ± 1.3 AUC, *P *=* *0.064). No significant effect was observed between the modalities at any other time‐point (pre *P *=* *0.292 and 1 h *P *=* *0.109).

### Collagen‐related peptide

When samples were incubated with xCRP (Fig. 1 panel C), significant differences in the MPA AUC occurred over time with participation in both RE (*P *=* *0.010) and CARE (*P *=* *0.005). Compared to pre‐exercise, MPAs were significantly increased immediately post‐RE (8.0 ± 2.4 AUC, *P *=* *0.012) and post‐CARE (10.5 ± 4.4 AUC, *P *=* *0.05). The decrease from immediately post to 1 h post‐exercise was significant following CARE (−11.6 AUC, *P *=* *0.012) but not post‐RE (−6.9 AUC, *P *=* *0.327). No changes were observed over time with no‐exercise (*P *=* *0.607) or aerobic exercise (*P *=* *0.687). No significant differences were found between the four modalities at corresponding time‐points (pre *P *=* *0.199, post *P *=* *0.154, and 1 h *P *=* *0.615).

## Discussion

Our aim was to compare the impacts of distinct forms of exercise on platelet function in overweight and physically inactive men. This is the first study to our knowledge in humans that has directly compared the acute impacts of routinely prescribed (Garber et al. 2011; Kang and Ratamess 2014) exercise modalities on monocyte‐platelet aggregation in the presence agonists of physiologically/clinically relevant pathways of platelet activation. We found that exercise increased agonist‐induced monocyte‐platelet aggregation immediately following exercise, with larger responses observed following modalities which included a resistance component (both RE and CARE). These effects of exercise on platelet function influenced all pathways of activation we studied, induced by TRAP, arachidonic acid, and xCRP. Finally, we observed a pattern of transient modification to MPA production, as immediate post‐exercise responses generally returned toward baseline levels by 1 h post‐exercise.

Arachidonic acid is a polyunsaturated omega‐6 fatty acid, which has diverse physiological functions that are ultimately dependent on its metabolic fate, as AA derivatives may be both pro‐ and anti‐inflammatory (Roman 2002). Among its many derivatives and their functions, the most relevant to the present investigation is AA's enzymatic conversion by cyclooxygenases to either prostacyclin (inhibitor of platelet activation and vasodilator) or thromboxane A2 (platelet activator and vasoconstrictor) (Needleman et al. 1986) and the non‐enzymatic conversion to isoprostanes (Morrow et al. 1990). While the mechanisms behind the metabolic outcomes of AA are complex, our data indicate that AA‐induced increases in MPAs are exercise modality dependent, with larger transient impacts of the RE and CARE conditions that both included a resistance exercise component. Arachidonic acid‐related effects on monocyte‐platelet aggregation have potential clinical significance, given the multiple roles of its downstream by‐products in terms of platelet aggregation in the atherothrombotic cascade (Frelinger et al. 2006; Praticò et al. 1997; Silver et al. 1973).

Thrombin is a central part of the coagulation cascade, produced by the prothrombinase complex following initiation of intrinsic or extrinsic coagulation. Thrombin activates platelets via cleavage of protease activated receptors, exposing their tethered ligand (Coughlin 2000; Giri and Jennings 2015). TRAP is the PAR‐1 ligand, and is commonly used as a platelet agonist in the interrogation of platelet function by flow cytometry (Vu et al. 1991). In keeping with the AA results above, we observed that MPAs transiently increased immediately following RE and CARE, and returned to near pre‐exercise levels by 1 h post‐exercise. No significant changes were observed in response to AE. Our finding that hypersensitivity to TRAP occurs following modalities of exercise involving a resistance component is of particular importance when considering acute exercise as a trigger of acute coronary syndromes, since tissue factor within atherosclerotic plaque is largely responsible for initiating thrombosis following atherosclerotic plaque rupture (Toschi et al. 1997; Wilcox et al. 1989)**.** Mechanistically, this finding indicates that increased sensitivity to platelet protease‐activated receptors occurs post‐exercise in response to RE and CARE.

Collagen is a component of the subendothelium which becomes exposed to flowing blood in the context of vascular injury. Collagen binds directly to two platelet surface receptors; integrin a2b1 and glycoprotein (GP) VI (Chen and López 2005). Collagen also binds to von Willebrand factor under shear, which binds to the GPIb‐IX‐V complex on platelets, resulting in vascular tethering and initiates signal transduction. Our data suggest that the propensity for increased monocyte‐platelet aggregation in response to collagen exposure is increased following RE and CARE, with smaller changes following AE. As in the case of AA and TRAP, this response was transient, returning to near pre‐exercise levels by 1 h post‐exercise.

Overall, the data presented above suggest that exercise involving a resistance component (i.e., RE, CARE) is more likely to sensitize platelets to activation and the formation of monocyte‐platelet aggregates than other exercise modalities (AE). There is some suggestion that exercise intensity may be a factor in differential regulation of platelet function (Hilberg et al. 2008). As steady‐state AE places continuous demand on the cardiorespiratory systems, whereas RE is intermittent, it is possible that the responses we found are not directly linked to cardiovascular stress, but rather linked to skeletal muscle. Indeed, if responses in this study are intensity dependent, it is evident that despite the relatively short duration of time actually spent exercising during RE, the influence on platelets may not be nullified by the recovery periods that separate exertional sets. With this in mind, it is important to acknowledge that it is not possible to match the intensities of steady‐state aerobic exercise and intermittent weight training without straying from the basic principles that characterize these contrasting modalities. The intention of the exercise interventions included in this study was to follow established guidelines for exercise prescription of these modalities ((ACSM) 2013; Garber et al. 2011), and adopt “real‐world” and relevant approaches. Our results are therefore ecologically valid and relevant to individuals participating in typical exercise modalities administered to the general population.

For each of the agonists we applied, there was a “general” pattern of transient increase in MPAs, with the highest values appearing immediately post‐exercise, followed by a return toward baseline levels. Our findings are in agreement with recent reviews and epidemiological evidence which suggests that increased atherothrombotic risk following acute exercise is transient in nature (Goodman et al. 2016; Riebe et al. 2015)**.** Evidence also indicates that acute exercise has the ability to cause rupture of thick fibrous cap atheroma that will not commonly rupture at rest (Tanaka et al. 2008), possibly due to increased heart rate, blood pressure, shear stress, and/or the concomitant impact of changes to the sympathetic nervous system and catecholamine levels during exercise (Birk et al. 2013; Gjovaag et al. 2015; MacDougall et al. 1985; Zouhal et al. 2008). In the context of the “exercise paradox”, it is possible that when individuals with endothelial dysfunction, arterial stiffness, and atherosclerosis participate in acute exercise, there is greater potential for the disruption of atheromatous plaque (fissure, erosion, rupture) or for vascular damage to occur, compared to physically active individuals with optimal vascular function and health (Birk et al. 2012; Falk et al. 2013; van Rosendael et al. 2015). Our findings suggest that, if plaque disruption and/or rupture occurs during or immediately post‐exercise, resulting in exposure of the pro‐thrombotic core to flowing blood (Wilcox et al. 1989), the consequent hyperthrombotic response may be more clinically relevant following resistive exercise bouts. Nonetheless, our data do not suggest that acute exercise is a direct stimulus that will consistently cause increases in platelet activation per se.

A potential limitation to this study is that we are unable to determine if responses are a result of the intensity of RE (i.e., to muscular failure) being greater than AE. Future research can approach this by investigating the impact of high‐intensity interval training using a common aerobic exercise modality on agonist‐induced MPAs. The purpose of this study was to compare typically utilized forms of exercise, that is, the forms and types of intervention commonly prescribed for middle‐aged men attending a gymnasium. We therefore selected the interventions based on observing the effects of an ecologically valid range of exercises. While it would be possible to match the exercise intensities between the conditions, this would result in spurious forms of exercise that bear little relevance to real world circumstances. Future research may also compare acute responses between different populations and training status, or before and after a long period of exercise training, to determine if the acute platelet function responses to a single bout of exercise is modulated. Finally, despite the present findings, it is important to acknowledge that the cardiovascular risk associated with acute exercise is small, and is far outweighed by the many benefits gained with chronic exercise training. In addition, the occurrence of ACS during exercise is lower in those who are regularly active (Siscovick et al. 1984), further emphasizing the importance of consistent participation in exercise to prevent such events.

In summary, we observed that, in the presence of TRAP, AA, and xCRP, exercise modalities involving resistance exercise evoked larger increases in platelet activation post‐exercise. This effect was transient and resiled following 1 h of recovery. In the context of recent studies suggesting that exercise may be capable of rupturing plaques that are not susceptible under resting conditions (Tanaka et al. 2008), these data suggest that modes of exercise involving a resistance component may transiently increase ACS risk more than typically administered forms of aerobic exercise.

## Conflict of Interest

None declared.
